# High yield expression of catalytically active USP18 (UBP43) using a Trigger Factor fusion system

**DOI:** 10.1186/1472-6750-12-56

**Published:** 2012-08-23

**Authors:** Anja Basters, Lars Ketscher, Elke Deuerling, Christoph Arkona, Jörg Rademann, Klaus-Peter Knobeloch, Günter Fritz

**Affiliations:** 1 Department of Neuropathology, University of Freiburg, Breisacher Str. 64, 79106, Freiburg, Germany; 2 Faculty of Biology, University of Freiburg, Schänzlestrasse 1, 79104, Freiburg, Germany; 3 Department of Biology, University of Konstanz, 78457, Constance, Germany; 4 University of Leipzig, Pharmazeutische Chemie, Brüderstraße 34, 04103, Leipzig, Germany

## Abstract

**Background:**

Covalent linkage of the ubiquitin-like protein ISG15 interferes with viral infection and USP18 is the major protease which specifically removes ISG15 from target proteins. Thus, boosting ISG15 modification by protease inhibition of USP18 might represent a new strategy to interfere with viral replication. However, so far no heterologous expression system was available to yield sufficient amounts of catalytically active protein for high-throughput based inhibitor screens.

**Results:**

High-level heterologous expression of USP18 was achieved by applying a chaperone-based fusion system in *E. coli*. Pure protein was obtained in a single-step on IMAC via a His_6_-tag. The USP18 fusion protein exhibited enzymatic activity towards cell derived ISG15 conjugated substrates and efficiently hydrolyzed ISG15-AMC. Specificity towards ISG15 was shown by covalent adduct formation with ISG15 vinyl sulfone but not with ubiquitin vinyl sulfone.

**Conclusion:**

The results presented here show that a chaperone fusion system can provide high yields of proteins that are difficult to express. The USP18 protein obtained here is suited to setup high-throughput small molecule inhibitor screens and forms the basis for detailed biochemical and structural characterization.

## Background

Posttranslational protein modification by ubiquitin and ubiquitin-like proteins (UBLs) serves as a versatile mechanism to control multiple biological functions in the cell [1]. The IFN-stimulated gene 15 (ISG15) is a UBL strongly induced by type I IFN and ISG15 conjugation (ISGylation) is one of the major antiviral effector systems [2-4]. Consequently, mice lacking ISG15 exhibit enhanced susceptibility upon distinct viral pathogens. Analogous to the ubiquitin modification process, conjugation of ISG15 is mediated by a cascade of E1, E2, and E3 ligases and ISG15 linkage is counteracted by the activity of deconjugating enzymes [5,6]. USP18 (UBP43) was shown to be the major ISG15 deconjugating enzyme and belongs to the peptidase C19 family [7]. As USP18 deficient mice and cells show elevated levels of ISG15- conjugated substrates [8], it appears feasible to enhance ISGylation levels by inhibition of the USP18 protease activity. This might also be of therapeutic interest as USP18 deficient animals were shown to be more resistant against certain viruses [3] and exhibit resistance against PML-RAR- [9] and BCR-ABL-induced leukemia [10]. A prerequisite for the identification of chemical compounds suitable to inhibit USP18 is the availability of a fast and sensitive enzymatic assay monitoring ISG15 deconjugation. High through-put screening based on ubiquitin-AMC (Ub-AMC) has been used with success for the identification of small molecules inhibiting USP protease activity. [11]. The assay is based on the release of the fluorophore AMC upon cleavage of the isopeptide bond by the USP. Thus, presumably ISG15 deconjugase inhibitors could be identified using ISG15-AMC in a similar protease inhibitor assay. However, in the case of USP18 the setup of such an assay for high-throughput screening was hampered so far by limited amounts of recombinant enzymatically active USP18. Attempts to express USP18 in *Escherichia coli (E. coli)* resulted in degraded protein [5]. Expression in Sf9 cells using the baculovirus expression systems was successful but is difficult to scale up and cost intensive [12] compared to bacterial expression systems. Here we report the development of a bacterial expression system based on the fusion of USP18 to a bacterial chaperone (Trigger Factor = TF) that yields high amounts of enzymatically active protein. USP18 was purified to homogeneity as a TF-fusion protein. The recombinant protease is specific for ISG15 as shown by enzymatic deconjugation of ISG15 from ISGylated cellular proteins and by the formation of a covalent adduct with ISG15 vinyl sulfone. Finally, we established assay conditions for USP18 mediated ISG15-AMC cleavage suited for the setup of large scale inhibitor screens.

## Methods

### Cloning methods

The consensus sequence for ubiqutinyl hydrolases encompasses residues 46–368 of USP18. The 45 N-terminal residues are of unknown function. cDNA encoding residues 46–368 of mouse USP18 in frame with a His_6_-tag and the recognition site for the 3 C protease was amplified and cloned into pET15b (Novagen) and pGEMEX (Promega) vector. All cloning steps were performed according to standard protocols [13].

For USP18 constructs with codons optimized for expression in *E. coli* a synthetic cDNA encoding USP18 residues 46–368, as well as a 3 C protease recognition site and a flexible linker at the 5’ end was purchased from a commercial supplier (Mr. Gene). cDNAs for His_6_-SUMO and His_6_-SUMO-TF_AAA_ were generated by PCR using vector pSUMO-tig_AAA_[14,15] as template. The cDNAs were fused and cloned into the vector pACE by sequence and ligation independent cloning (SLIC) [16] yielding vectors pACE-His_6_-SUMO-TF_AAA_-USP18 and pACE-His_6_-SUMO-USP18. NdeI and XhoI restriction sites were inserted to allow further subcloning of the constructs.

The following primers were used for vector and insert amplification and ligation: XhoI-pACE-for: 5’-CTCGAGAGATCCGGCTGCTAACAAAG-3’, NdeI-pACE-rev: 5’-CATATGTATATCTCCTTCTTAAAGTTAAAC-3’, XhoI-USP18-rev: 5’-CTTTGTTAGCAGCCGGATCTCTCGAGTTAGGAGCCGGTTTTCG-3’,   SUMO-3C-for:  5’-CACAGAGAACAGATTGGTGGTCTGGAAGTTCTGTTCCAGGGTCCG-3’,  TF-for: 5’-CACTTTCAACGAGCTGATGAACCAGCAGGC-3’, TF-rev: 5’-GCCTGCTGGTTCATCAGCTCGTTGAAAGTG-3’,  SUMO-3C-rev:  5’-CGGACCCTGGAACAGAACTTCCAGACCACCAATCTGTTCTCTGTG-3’, NdeI-His-for: 5’-GTTTAACTTTAAGAAGGAGATATACATATGATGGGTCATCACCATCATC-3’.

For cloning into the pSUMO backbone [14,15], the pACE expression vectors were digested with NdeI and XhoI restriction enzymes and the inserts were ligated into pSUMO vector digested with the same enzymes. A catalytic inactive mutant with substitution of the catalytic cysteine 61 to alanine was generated using the QuikChange II kit (Stratagene).

### Expression and purification

The following strains were transformed with the different vectors and tested for expression: *E. coli* BL21(DE3), *E. coli* BL21(DE3)pLysS, *E. coli* Rosetta(DE3) , *E. coli* Tuner(DE3) and *E .coli* Tuner(DE3)pLysS (Novagen). Expression was performed in shaking cultures in DYT medium supplemented with appropriate antibiotics, trace elements (Studier) and 0.2% (w/v) glucose. For *E. coli* strains transformed with pET15b, pGEMEX or pACE 100 μg/ml ampicillin was added to the medium; for pSUMO 50 μg/ml kanamycin was added to the medium. In case of *E. coli* strains *E. coli* Rosetta(DE3), *E. coli* BL21(DE3)pLysS and *E. coli* Tuner(DE3)pLysS additionally 17 μg/ml choramphenicol was added to the medium.

5 ml DYT medium was inoculated with a single colony and incubated on a shaker at 37°C overnight. For inoculation of expression cultures the overnight culture was diluted 1:100 in the same medium. Test expression cultures had a volume of 20 ml in 200 ml Erlenmeyer flasks at different temperatures (15°C - 37°C). The culture was grown until an OD_600 nm_ of 0.6 was reached and expression was induced by addition of IPTG (Applichem) to a final concentration of 0.1 - 1 mM. Large scale expression was performed in 500 ml in baffled 2 l Erlenmeyer flasks at 15°C for 16 h with a final IPTG concentration of 0.1 mM.

Cells from expression cultures were harvested by centrifugation. Cell pellets were suspended in ice-cold buffer A (20 mM Tris-Cl, 500 mM NaCl, pH 7.9). Cell pellets from small scale expression were disrupted by ultrasonic treatment whereas cells from large scale expression were broken by 2 passages through a French pressure cell at 137 Mpa. Typically, 8 g wet weight cells were used per batch of protein purification.

Crude extracts from test expressions were centrifuged at 4°C at 16,000 g for 60 minutes. Supernatant and pellet fraction were mixed with sample buffer and analyzed on SDS-PAGE. Crude extracts from large scale expression were centrifuged at 100,000 g for 60 min. All purification steps were performed at 4°C using a FPLC system (GE-Healthcare). The supernatant was applied to a Cobalt-IMAC column (1 ml column volume, Novagen) equilibrated in buffer A. Absorption at 280 nm was monitored and column was washed with the same buffer until absorption reached baseline level again. Three washing steps were performed with buffer A supplemented with 10 mM, 20 mM and 30 mM imidazole, respectively. The bound protein was eluted with buffer A containing 1 M imidazole. The pure protein was dialyzed against 5 l buffer A overnight, concentrated to 8 mg/ml and analyzed by SDS-PAGE and subsequent Coomassie staining.

### Generation of ISGylated cell lysates and deISGylation assay

USP18 deficient murine embryonic fibroblasts (MEFs) were stimulated with 250 U/ml IFN β (Sigma) for 24 h to induce ISGylation of endogenous proteins or left untreated. MEFs were lysed in 50 mM Tris-Cl pH 7.4, 150 mM NaCl, 1 mM EDTA, 1% Triton X-100. The cell lysate was cleared by centrifugation at 16,000 g for 30 min at 4°C. 5 μl of the supernatant (20 μg) were incubated with 2 μl (16 μg) TF_AAA_-USP18 and 20 μl reaction buffer (50 mM Tris-Cl pH 8.3, 25 mM KCl, 5 mM MgCl_2_, 1 mM DTT) for 0, 1 and 2 h at 37°C. The reaction was stopped by addition of SDS containing sample buffer. The samples were separated on a 12% SDS-PAGE gel, transferred to a nitrocellulose membrane and analyzed with the following antibodies: ISG15 [17] and β-Actin (I-19, Santa Cruz). For quantification the optical densities of protein bands were obtained using ImageJ [18]. The densitometric values of free and conjugated ISG15 were normalized to β-Actin and depicted relative to the ISG15 values in IFN β-treated cells at 0 h without addition of TF_AAA_-USP18.

### Reaction with ubiquitin and ISG15 vinyl sulfone

HA-Ubiquitin (Ub-VS) and HA-ISG15 vinyl sulfone (ISG15-VS) were purchased from Boston Biochem. 1 μl (8 μg) of TF_AAA_-USP18 or TF_AAA_-USP18-C61A was combined with 1 μl (0.5 μg) Ub-VS or ISG15-VS, respectively. Reaction was performed in 50 mM Tris-Cl pH 7.4, 5 mM MgCl_2_, 250 mM sucrose, 1 mM DTT, 2 mM ATP for 1 h at 37°C [19]. The samples were analysed on a Coomassie-stained 10% SDS-PAGE gel.

### Measurement of ISG15-AMC cleavage

ISG15-amidomethyl coumarin (AMC) was purchased from Boston Biochem. Different amounts of TF_AAA_-USP18 (final concentration 0, 0.36, 0.72, 1.43 μM) were incubated with 600 nM ISG15-AMC in a total volume of 28 μl. For each TF_AAA_-USP18 concentration duplicates were analyzed. The reaction was performed in 50 mM Hepes-NaOH pH 7.5, 0.01% (v/v) Tween 20, 10 mM DTT. The release of AMC was detected over a period of 30 minutes using a Safire II fluorescence spectrophoto meter with excitation and emission wavelength of 380 nm and 460 nm, respectively.

## Results and discussion

High-throughput screening requires large amounts of active protein. Recombinant expression of USP18 in a bacterial system as well as in insect cells has been reported, however with very low yields [5]. Until now, there has been no expression system available for production of sufficient amounts of recombinant USP18. Therefore, we aimed to establish a high-yield and easy-to-apply expression system for catalytically active USP18.

**Figure 1:**
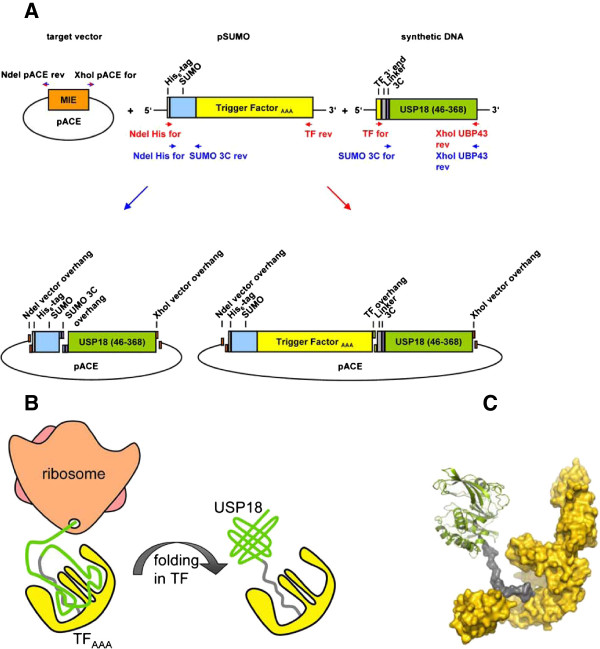
(**A**) **Generation of SUMO-USP18 and SUMO-TF**_**AAA**_**-USP18 expression vectors using sequence and ligation independent cloning (SLIC). **The target vector pACE was linearized using primers NdeI-pACE-rev and XhoI-pACE-for. Recognition sites for the restriction enzymes NdeI and XhoI were introduced during the amplification process. Two vectors served as templates for amplification of the inserts: pSUMO encoding a His_6_-tag-SUMO-Trigger Factor_AAA_ fusion protein, and a synthetic DNA construct consisting of the 3’ end of the Trigger Factor, a flexible linker, a recognition site for the 3 C protease and a USP18 cDNA for residues 46–368. For both the SUMO-USP18 and the SUMO-TF_AAA_-USP18 construct, two PCR products with overlapping 5’ and 3’ ends were generated. For the SUMO-USP18 construct primers NdeI-His-for together with SUMO-3C-rev as well as primers SUMO-3C-for together with XhoI-USP18-rev (blue) were used. For the SUMO-TF_AAA_-USP18 construct, primer NdeI-His-for was combined with TF-rev and primer TF-for with XhoI-USP18-rev (red). Treatment of the PCR products with T4 DNA polymerase in the absence of dNTPs resulted in complementary single stranded overhangs that were subsequently annealed. The resulting vectors encoded for two different USP18 fusion proteins: SUMO-USP18 and SUMO-TF_AAA_-USP18. Both proteins exhibit a His_6_-tag for purification and a SUMO-tag to enhance solubility of the protein. In the SUMO-TF_AAA_-USP18 protein the bacterial chaperone Trigger Factor carrying three exchanges to alanine (F43A, R44A, K45A; =TF_AAA_) is additionally fused to the N-terminus of USP18 to provide each expressed USP18 molecule a chaperone that facilitates folding. The flexible linker between TF_AAA_ and USP18 was introduced to allow interaction of USP18 with the chaperone. (**B**) Schematic drawing of TF_AAA_ -USP18. Newly synthesized TF_AAA_ folds and takes up the nascent chain of USP18. The fusion protein dissociates from the ribosome and USP18 can fold in the cradle of TF_AAA_. (**C**) Molecular model of TF_AAA_-USP18. TF_AAA_ is shown as surface representation and USP18 as cartoon with secondary structure elements. TF_AAA_ is shown in yellow, the linker in grey, and USP18 in green, respectively.

Expression trials using murine cDNA for USP18 cloned into pET15b or a pGEMEX vector were performed in *E. coli* Rosetta(DE3). However, no expression of His_6_-tagged USP18 could be observed in Western blots (Table 1). We reasoned that some rare codons in the cDNA of the USP18 clone might obstruct expression and therefore switched to an expression construct with codons optimized for expression in *E. coli*. In addition, we introduced a SUMO-tag at the N-terminus of USP18 as such a tag was reported to enhance expression levels of this protein in the baculovirus expression system [12]. Sequence and ligation independent cloning (SLIC) was performed to generate the His_6_-SUMO-USP18 construct in the pACE vector backbone [20,21] (Figure 1A). Subsequently, *E. coli* BL21(DE3) as well as *E. coli* BL21(DE3)pLysS were used as host strains for test expressions. In contrast to the clone derived from mouse cDNA, expression of the SUMO-USP18 fusion protein could be detected in both *E. coli* strains on Western blot with an anti-His_6_-tag specific antibody. However, expression levels of the fusion protein were too low to be detected on Coomassie-stained SDS-PAGE gel (Table 1).

**Table 1:** Constructs tested for expression of different USP18 fusion proteins

**Construct (promoter, antibiotic resistance)**	**pGEMEX-His-USP18 (T7, ampicillin)**	**pET15b-His-USP18 (T7, ampicillin)**		**pACE-His**_**6**_**-SUMO-USP18 (T7, ampicillin)**		**pACE-His**_**6**_**-SUMO-TF**_**AAA**_**-USP18 (T7, ampicillin)**	
**strain**	**Rosetta(DE3)**	**Rosetta(DE3)**		**BL21(DE3)**	**BL21(DE3)pLysS**	**BL21(DE3)**	**BL21(DE3)pLysS**
temperature	25°C	25°C	37°C	37°C	25°C	37°C	37°C
expression	-	-	-	+	+	++	++
soluble	n.d.	n.d.	n.d.	n.d.	n.d.	n.d.	n.d.
**Construct (promoter, antibiotic resistance)**	**pSUMO-His-SUMO-TF_AAA_-USP18 (T7, kanamycin)**						
**strain**	**BL21(DE3)**		**BL21(DE3)pLysS**			**Tuner(DE3)pLysS**	**Tuner(DE3)**
temperature	25°C	37°C	15°C	25°C	37°C	15°C	15°C
expression	+++	+++	+	+++	+++	++	+++
soluble	n.d.	n.d.	+	-	-	++	+

n.d.: not determined, -: no expression observed, +: detectable on Western Blot, but not on Coomassie-stained SDS PAGE gel, ++: detectable on Coomassie-stained gel, but not the dominant band, +++: dominant band on Coomassie-stained gel.

Overproduction of soluble recombinant protein in *E. coli* can be limited by the deprivation of host cell chaperones that are required for correct folding of the respective protein. Co-overexpression of *E. coli* chaperones was reported previously to enhance solubility and yield of recombinant proteins [22]. Recently, also successful expression of a fusion of the chaperone Trigger Factor with the protein of interest was reported using a cold shock expression system in *E. coli*[23] (Takara, pCold TF plasmid). This system provides each translated protein its own chaperone. As the chaperone Trigger Factor (TF) is the first chaperone newly translated proteins encounter [24] we fused this chaperone to the N-terminus of USP18. TF interacts with the bacterial ribosome and incorporates nascent polypeptide chains that emerge from the ribosomal exit tunnel. In this way, it provides a protective environment that facilitates folding [24,25]. To assure interaction of USP18 with TF, which forms a large hydrophobic cradle, we introduced a long flexible linker consisting of six GSS repeats between USP18 and the chaperone (Figure 1B, C). Moreover, the long linker ensures that the folded USP18 is accessible for substrates and not sterically blocked by TF.

TF binds to the ribosome via the motif 43-GFRxGxxP-50 [26,27]. Although TF binds with low affinity to the ribosome [28], overexpression of TF might become a serious problem for protein synthesis in the expression host. In order to reduce binding of the TF-USP18 fusion protein to the ribosome and facilitate dissociation, residues G43, F44 and R45 of TF were exchanged to alanine (TF_AAA_). These residues have been shown previously to be critical for TF-ribosome interaction [27].

The resulting fusion protein consists of an N-terminal His_6_-tag, SUMO, Trigger Factor_AAA_, and USP18 (= TF_AAA_-USP18). TF_AAA_-USP18 in the pACE vector backbone was tested for expression in *E. coli* BL21 (DE3) and *E. coli* BL21 (DE3) pLysS. In contrast to SUMO-USP18, insertion of TF increased expression levels so that the fusion protein could be detected on Coomassie-stained SDS-PAGE gel (Table 1). However, it did not represent the major fraction compared to endogenous bacterial proteins.

**Figure 2:**
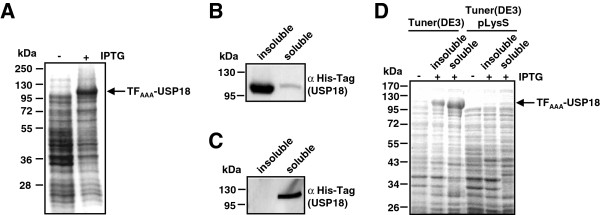
**Expression of TF**_**AAA**_**-USP18 in pSUMO vector backbone under different conditions(A) TF**_**AAA**_**-USP18 was expressed in E. coli BL21(DE3)pLysS at 37°C. **Expression was verified by analyzing protein content directly after lysis on SDS-PAGE followed by Coomassie staining. After 3 h of induction TF_AAA_-USP18 fusion protein made up more than 50% of whole cellular proteins. (**B**) Soluble and insoluble fractions from (A) were analysed by Western blot with an anti His_6_-Tag antibody. Almost all fusion protein was present in the insoluble fraction and only a faint band for soluble protein was observed. (**C**) Expression of TF_AAA_-USP18 at 15°C in *E. coli* BL21(DE3)pLysS yielded soluble protein. Western blot analysis using an anti His_6_-Tag antibody detected TF_AAA_-USP18 only in the soluble fraction. (**D**) *E. coli* Tuner(DE3) and *E. coli* Tuner(DE3)pLysS were tested for expression of TF_AAA_-USP18 at 15°C. Soluble and insoluble fractions were analysed by SDS-PAGE and subsequent Coomassie staining. Strong expression was only observed in *E. coli* Tuner(DE3). The major portion of the fusion protein was observed in the soluble fraction.

Therefore, we changed the vector backbone from pACE to pSUMO. This boosted expression of the fusion protein which now represented the major band on SDS gel when expression was performed at 37°C (Table 1 and Figure 2A). However, these expression conditions resulted in poor solubility of the protein as demonstrated by Western Blot with a His_6_-tag specific antibody (Figure 2B). Almost all recombinant protein was detected in the pellet fraction whereas only a weak band was detected in the soluble fraction. Lowering temperature is often reported to increase yield and solubility of expressed proteins [29-31]. Test expressions at 25°C had no observable effect and resulted in insoluble protein (not shown). Decreasing further the expression temperature to 15°C yielded soluble TF_AAA_-USP18 (Figure 2C). However, the drop in temperature caused also a severe decrease in protein expression (Table 1).

**Figure 3:**
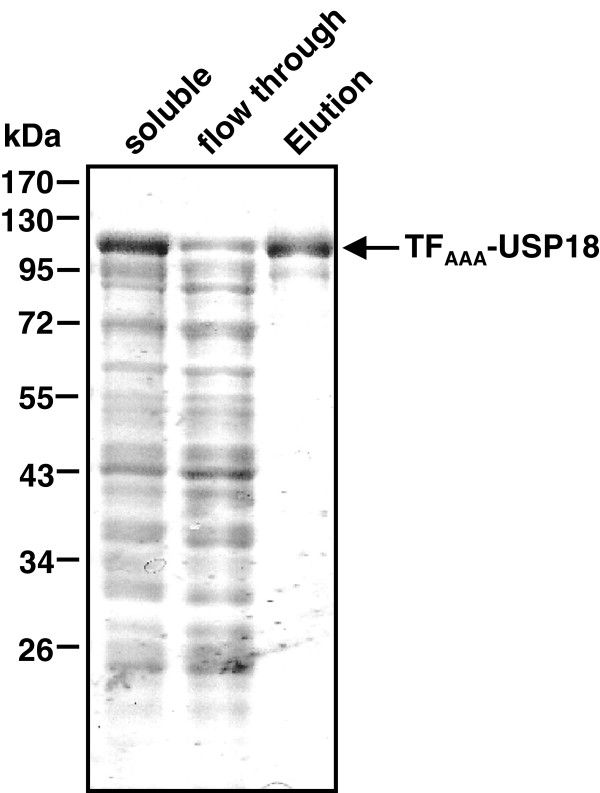
**One-step purification of TF**_**AAA**_**-USP18. **TF_AAA_-USP18 was bound to a Co-IMAC column and eluted with imidazole. Purity of the eluted fusion protein was visualized by SDS-PAGE with subsequent Coomassie staining.

To achieve again high expression levels combined with high solubility of TF_AAA_-USP18 we changed to the stringent expression host strains *E. coli* Tuner(DE3) and *E. coli* Tuner(DE3)pLysS. Tuner strains are deficient in lactose permease (*lacY*) and thus allow uniform uptake of IPTG via diffusion. Whereas *E. coli* Tuner(DE3)pLysS only showed a weak expression of TF_AAA_-USP18, strong expression of soluble fusion protein was observed when the *E. coli* Tuner(DE3) strain was grown at 15°C (Table 1 and Figure 2D). Therefore, these conditions were applied for large scale expression and purification. 2 liter expression cultures typically yielded 24 g of wet weight pellet. For purification of TF_AAA_-USP18, different IMAC columns were tested of which a cobalt IMAC column provided the best results. Using this column, pure TF_AAA_-USP18 was eluted allowing one-step purification without need of further purification steps (Figure 3). A minor band running at lower molecular weight was observed when the sample was boiled only for a short time or without fresh DTT added. This band most likely represents protein containing an intramolecular disulfide bond formed during boiling. Typical yield was 10 mg pure protein out of 8 g wet weight pellet.

**Figure 4:**
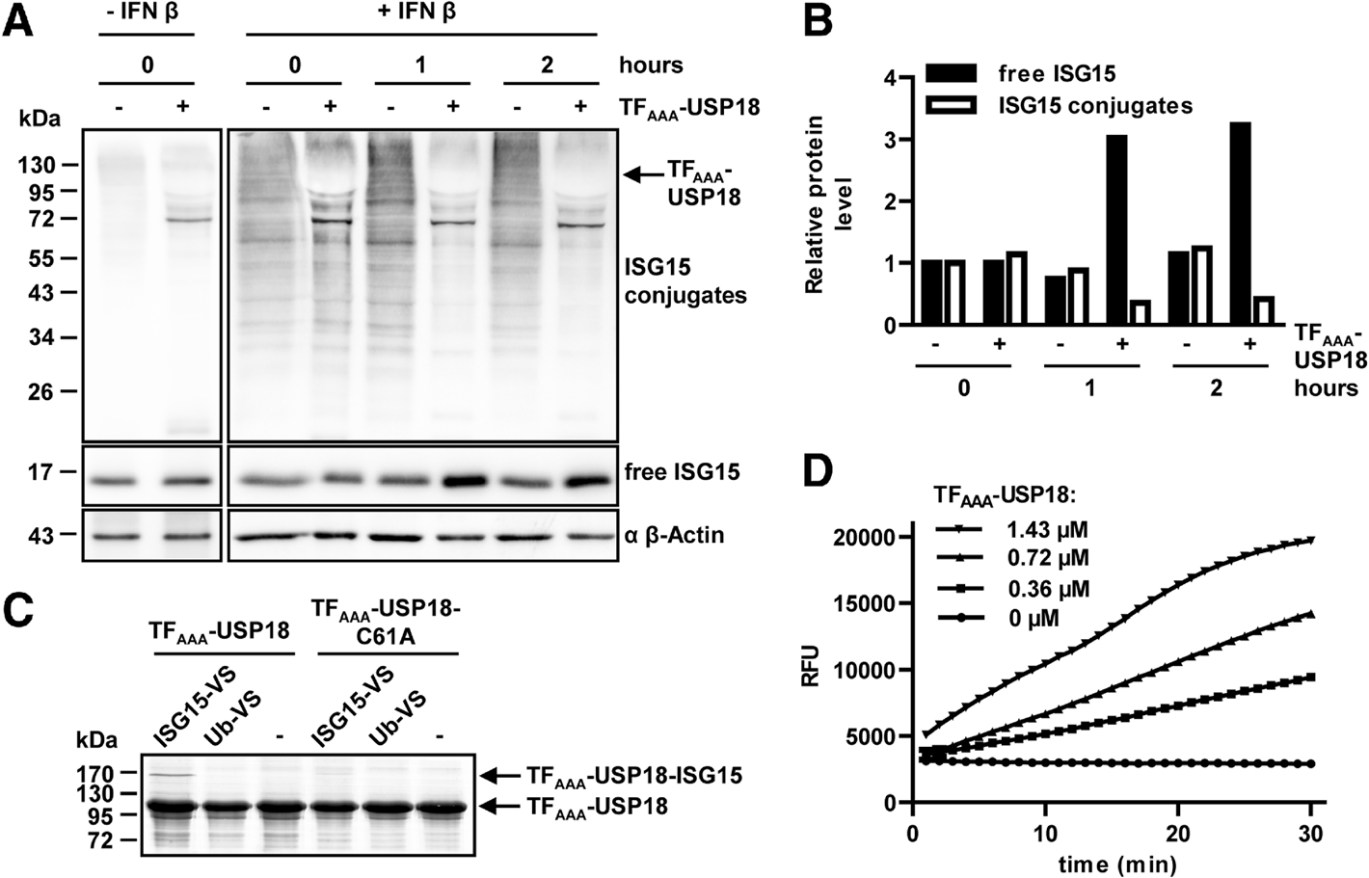
**Enzymatic activity of TF**_**AAA**_**-USP18 (A) Cell lysates of USP18 knockout mouse embryonic fibroblasts were stimulated with IFN β resulting in elevated ISGylation or left untreated. **Cell lysates were incubated with and without TF_AAA_-USP18 for the indicated times at 37°C. TF_AAA_-USP18-mediated ISG15 deconjugation was monitored by Western blot with an ISG15-specific antibody. A decrease of protein ISGylation with a concomitant increase of free ISG15 was observed. (**B**) Quantification of ISGylation and free ISG15 from (**A**) revealed a 3-fold decrease of conjugated and a corresponding increase of free ISG15 upon incubation with TF_AAA_-USP18. Densitometric values of free ISG15 and ISGylated proteins were normalized to β-Actin and to the protein levels in IFN β-treated cells at 0 hours without addition of TF_AAA_-USP18. (**C**) TF_AAA_-USP18 or the catalytically inactive mutant TF_AAA_-USP18-C61A were incubated with substoichiometric amounts of either ISG15 vinyl sulfone (ISG15-VS) or ubiquitin vinyl sulfone (Ub-VS) for 1 h at 37°C. The covalent adduct of USP18 with ISG15 is shown by a shift to higher molecular mass visualized on a Coomassie-stained SDS PAGE. (**D**) Catalytic activity of TF_AAA_-USP18 monitored by cleavage of ISG15-AMC: different amounts of TF_AAA_-USP18 were incubated with 600 nM ISG15-AMC. Release of AMC was monitored by its specific fluorescence at 460 nm over a period of 30 minutes. RFU: Relative fluorescence units.

Once expression and purification was established we checked whether the large scale preparations represent also catalytically active enzyme. Therefore, we tested isopeptidase activity of TF_AAA_-USP18 towards ISG15 modified cellular proteins (Figure 4A). High levels of ISGylated cellular protein were obtained using USP18 deficient mouse embryonic fibroblasts (MEFs) stimulated with IFN β. MEF cell lysates were incubated with and without TF_AAA_-USP18 and changes in ISGylation levels were monitored by Western blot with an ISG15-specific antibody. Incubation with TF_AAA_-USP18 drastically decreased the amount of ISGylated proteins, simultaneously the amount of free ISG15 increased demonstrating the ability of the TF_AAA_-USP18 to recognize and cleave ISG15 from cellular target proteins. To further evaluate enzymatic specificity, TF_AAA_-USP18 as well as a TF_AAA_-USP18 variant, where the catalytic cysteine is exchanged to alanine (TF_AAA_-USP18-C61A), were incubated with the suicide inhibitors ubiquitin vinyl sulfone (Ub-VS) and ISG15 vinyl sulfone (ISG15-VS), respectively. These suicide inhibitors form a covalent adduct upon reaction with the active site cysteine of ubiquitin-specific proteases. The reaction can be visualized as a shift in molecular mass on a Coomassie-stained gel. For TF_AAA_-USP18, covalent complex formation was detected with ISG15-VS whereas mutation of the catalytic cysteine to alanine resulted in complete loss of the interaction. Neither TF_AAA_-USP18 nor TF_AAA_-USP18-C61A showed cross-reactivity towards Ub-VS (Figure 4B). In summary, these experiments show that TF_AAA_-USP18 is catalytically active and underline its specificity towards ISG15.

Screening for potential USP18 inhibitors requires a method that allows quantification of USP18 activity and is compatible with standard detection instruments. Therefore, we established assay conditions for TF_AAA_-USP18-mediated ISG15-AMC cleavage. Different amounts of the fusion protein were incubated with ISG15-AMC and cleavage was measured over a period of 30 minutes. The measured rate of ISG15-AMC cleavage was constant for more than 20 minutes and the rate increased linearly with enzyme concentration (Figure 4C). At the highest concentration of TF_AAA_-USP18, a slight decrease in the rate was observed after 25 minutes that is most likely due to a limitation of substrate and not caused by a decrease in enzyme activity. TF itself has no isopeptidase catalytic activity and does not interfere with the assay. These results demonstrate that TF_AAA_-USP18 is very well suited for kinetic analysis and the assay presented here can be easily adapted for high-throughput screening for specific inhibitors of USP18.

## Conclusion

Today, the analysis of genomic and expressions array data provide a plethora of data on proteins in the regulation of vital cellular processes representing potential therapeutic targets. The entire process of drug development relies on the availability of correctly folded and active target proteins provided by heterologous production in eukaryotic and prokaryotic expression systems. However, for fast, efficient, and easy-to-scale-up expression *E. coli* is still the expression system of choice. Here, we describe a new and efficient method to express and purify high yields of recombinant catalytically active USP18. Starting from zero expression we could boost the yields of active protein by optimization of codons, vector backbone, and a novel chaperone fusion system. The excellent yields obtained for USP18 put forward that this system is also very well suited for other proteins where recombinant expression failed so far.

## Abbreviations

ISG: IFN-stimulated gene; USP: Ubiquitin-specific protease; HA: Hemagglutinin; AMC: 7-amino-4-methylcoumarin; IFN β: Interferon β.

## Competing interest

The authors declare that they have no competing interests.

## Authors’ contributions

AB carried out the cloning of vectors, performed expression, purification, and analysis of proteins, and wrote the manuscript. LK carried out design and cloning of expression vectors. ED designed expression vector and expression experiments. CA and JR have conducted assays with USP18 using ISG-15-AMC as a substrate. KPK designed expression experiments and activity assay, and wrote the manuscript. GF designed expression vectors and experiments, purified protein and wrote the manuscript. All authors read and approved the final manuscript.
